# Exploring the concept and management strategies of caring stress among clinical nurses: a scoping review

**DOI:** 10.3389/fpsyt.2024.1337938

**Published:** 2024-05-28

**Authors:** Amir Hossein Goudarzian, Alireza Nikbakht Nasrabadi, Hamid Sharif-Nia, Bahar Farhadi, Elham Navab

**Affiliations:** ^1^ Department of Psychiatric Nursing, School of nursing and midwifery, Tehran University of Medical Science, Tehran, Iran; ^2^ Student Research Committee, Mazandaran University of Medical Sciences, Sari, Iran; ^3^ Department of Medical-Surgical Nursing and Basic Sciences, School of Nursing & Midwifery, Tehran University of Medical Sciences, Tehran, Iran; ^4^ Psychosomatic Research Center, Mazandaran University of Medical Sciences, Sari, Iran; ^5^ Department of Nursing, Amol Faculty of Nursing and Midwifery, Mazandaran University of Medical Sciences, Sari, Iran; ^6^ School of Medicine, Islamic Azad University, Mashhad Branch, Mashhad, Iran; ^7^ Department of Critical Care and Geriatric Nursing, School of Nursing & Midwifery, Tehran University of Medical Sciences, Tehran, Iran

**Keywords:** care, stress, workload, nurse, emotions, mental health

## Abstract

**Objective:**

The concept of caring stress and its specific management has received less attention than other dimensions of stress in nurses. Therefore, to clarify the concept of caring stress, a scoping review study was designed. This scoping review aimed to investigate the concept of caring stress among clinical nurses and examine the strategies used for its management.

**Methods:**

This review employed a scoping review methodology to comprehensively map the essential concepts and attributes of the phenomenon by drawing on a wide range of sources. International databases including PubMed, Scopus, Web of Science, Google Scholar, and Scientific Information Database (SID) were searched to gather relevant studies published until October 1, 2023. MESH terms included “caring stress”, “care”, “stress”, “nurse”, and “stress management” were used. Two reviewers independently collected data from full-text papers, ensuring that each paper underwent assessment by at least two reviewers.

**Results:**

Out of 104,094 articles initially searched, 22 articles were included in this study. High workloads, transmitting the infection, stressful thoughts, stressful emotions, and stressful communications were the significant concepts and factors of caring stress among nurses. Also, rest breaks during patient care shifts, playing music in the ward, and denial of critical situations were examples of positive and negative coping and management ways to reduce caring stress.

**Conclusion:**

Effective stress management strategies can lead to better patient care and safety. Stressed nurses are more likely to make errors or become less vigilant in their duties, impacting patient outcomes. By addressing caring stress, clinical practice can prioritize patient well-being. Further research is required to delve deeper into this critical issue concerning nurses in the future.

## 1 Introduction

Nursing is a stressful profession that can affect both the physical and mental health of nurses. This stress stems from dealing with difficult and emotionally challenging situations, such as caring for critically ill patients, managing family emotions, and heavy workloads (1). Stress impacts nursing performance, job satisfaction, and patient outcomes.

Nursing is a very stressful profession worldwide, with high rates of emotional, mental, and physical injury, and job dissatisfaction (2). Over 50% of nurses experience high stress levels at work, making it more stressful than other healthcare professions because of their direct involvement in patient care (3).

Stress can have serious effects on nurses, both physically and mentally. It can cause health issues like high blood pressure and depression, leading to absenteeism and reduced productivity (2). Research shows that stressed nurses are more likely to make mistakes and decrease patient satisfaction. It is crucial to address stress in nursing to improve patient care and nursing performance (4, 5).

The stress concepts discussed so far have covered all stressors in the nursing job environment including occupational stress, environmental stress, and mental or emotional stress (2). While occupational stress may stem from workload demands and administrative pressures, caring stress primarily emerges from the emotional toll of empathizing with patients’ suffering and witnessing their struggles firsthand (6). Unlike environmental stressors that may arise from the physical conditions of the workplace, caring stress manifests from the intense interpersonal dynamics inherent in nursing care.

Additionally, while mental or emotional stress can result from personal life challenges, caring stress is deeply rooted in the professional realm, often triggered by the ethical dilemmas, moral distress, and emotional fatigue that nurses encounter while providing patient care (7). Caring stress underscores the unique burden borne by nurses as they navigate the delicate balance between compassion and self-care in their demanding roles (8). However, the core principle of the nursing profession revolves around patient care, and the notion of caring stress specifically encompasses stressors related to this aspect.

Based on available databases, the prevalence of caregiver stress has not yet been assessed. Nurses often face caring stress for various reasons, such as direct interaction with patients and their families, managing multiple patients simultaneously, and dealing with the unstable conditions of hospitalized patients (9, 10). The persistence and chronicity of caring stress can lead to physical consequences, including increased blood pressure, cardiovascular diseases, and sleep disturbances, as well as psychological effects, such as depression and anxiety. Ultimately, it affects the nurse’s quality of life and the quality of patient care (11).

Nurses experience caring stress due to complex patient cases, ethical dilemmas, emotional and psychological burdens, rapid decision-making, and responsibility for patient outcomes. Stress is a constant presence in their daily work (12). Caring stress extends into communication challenges, the scope of practice, and the advocacy of patient rights, presenting an array of challenges that nurses navigate daily (13).

One of the best ways to prevent or mitigate the mentioned consequences is the management of caring stress in nurses (4, 14). Caring stress management is considered one of the methods to achieve happiness and job satisfaction in the social and professional context. It means that nurses, through better stress management and adherence to psychological health principles, create the best conditions for happiness and job satisfaction (15). The management of caring stress can be viewed (from a philosophical standpoint) as both an individual and social process situated within the societal value system. For example, in Aristotle’s philosophy, “happiness” is regarded as the ultimate goal of human life (16). In this sense, happiness includes satisfaction, joy, and a sense of both spiritual and physical well-being (17).

So, the management of caring stress is regarded as a pathway toward attaining happiness, job satisfaction, and overall social well-being. It means that nurses, through better stress management and adherence to psychological health principles, create the best conditions for increasing mental health and job satisfaction (10, 15). Therefore, from a philosophical perspective, the management of caring stress is considered a key concept and process in achieving happiness, job satisfaction, peace, and purpose in the lives of individuals (18).

In the realm of nursing, the concept of caring stress management signifies the adoption of strategies and approaches designed to mitigate and regulate professional stress within the nursing work environment (19). So far, several systematic reviews have been carried out on stress in nursing (20–22). Most studies on nursing stress have focused on job stress related to factors like shift changes, high workload, and staff shortages. However, a universal source of stress for nurses worldwide is caring for patients. This type of stress, known as caring stress, involves the challenges and stressors nurses face while delivering direct patient care. Despite its importance, this aspect has not been thoroughly researched by scholars (23).

Furthermore, researchers have primarily focused on reviewing stress-reducing interventions for managing caring stress (24, 25). However, since most researchers have traditionally focused on occupational stress (20, 22), it is necessary to shift the approach and give more attention to caring stress, as well as exploring methods for managing it. This scoping review study will be the first to examine management aspects based on caring stress.

A scoping review method has been chosen as a rapid review approach (26) to expedite the synthesis of existing research evidence. Moreover, we have chosen to conduct a scoping review based on our initial investigations, which have revealed the considerable breadth of the subject area being studied. Therefore, a scoping review will aid in refining the pool of available studies by conducting a comprehensive and systematic literature search (26). This search will serve as a foundational resource for potential future systematic reviews, specifically tailored to address more precise inquiries, such as the prevalence of caring stress, its underlying reasons, and other aspects relevant to developing a comprehensive assessment scale for this concept among nurses.

The concept of caring stress and its specific management has received less attention compared to other dimensions of stress in nurses. Therefore, to clarify the concept of caring stress, a scoping review study was designed. This review study is essential for tackling the urgent issue of caring stress within the nursing profession. It has far-reaching implications for nurse well-being, patient care quality, and the sustainability of the nursing workforce. By summarizing the latest evidence-based practices and offering guidance for healthcare policies and leadership, this study can contribute to a more compassionate and effective healthcare system.

## 2 Methods

### 2.1 Study design

This review employed a scoping review methodology to comprehensively map the essential concepts and attributes of the phenomenon by drawing on a wide range of sources (27). The review followed the Joanna Briggs Institute (JBI) methodology for scoping reviews, with further refinements made in accordance with the guidelines outlined by Peters, Godfrey (28). It also followed the Preferred Reporting Items for Systematic Reviews and Meta-Analyses extension for Scoping Reviews (PRISMA-ScR) to ensure transparency and rigor in the reporting process (29).

### 2.2 Review question(s)

The primary goal and its associated subsidiary inquiries served as the guiding framework for this review. The fundamental objective of this scoping review was to aggregate and visually depict the findings reported in the global literature about caring stress and its management. Caring stress, operationally defined, represents a specific form of stress experienced by nurses in connection with their patient care duties (more concise than occupational stress).

The following are the sub-questions that constitute the specific objectives of this study:

- What are the aspects and reasons of caring stress in nurses?
- What are the aspects of caring stress management in nurses?

Moreover, the PICO format specified the population as nurses, the intervention as management strategies for dealing with caring stress, and the outcome as understanding the concept of caring stress and identifying effective management strategies for nurses.

### 2.3 Search strategy and selection criteria

We searched international databases, including PubMed, Scopus, Web of Science, Google Scholar, and the Scientific Information Database (SID), to gather relevant studies published until October 1, 2023. MESH terms such as “caring stress,” “care,” “stress,” “nurse,” and “stress management,” were utilized with the use of ‘OR’ and ‘AND’ operators for evaluating the selected databases. Details regarding the search strategy are presented in **Table 1**.

**Table 1:** Searching procedure.

Databases	Search strategy	Preliminary search	First screening^*^	Second screening^**^
**PubMed**	(“care stress”[All Fields] OR “care”[All Fields] OR “stress”[All Fields]) AND “nurse”[All Fields] AND ((“stress”[All Fields] OR “stressed”[All Fields] OR “stresses”[All Fields] OR “stressful”[All Fields] OR “stressfulness”[All Fields] OR “stressing”[All Fields]) AND (“manage”[All Fields] OR “managed”[All Fields] OR “management s”[All Fields] OR “managements”[All Fields] OR “manager”[All Fields] OR “manager s”[All Fields] OR “managers”[All Fields] OR “manages”[All Fields] OR “managing”[All Fields] OR “managment”[All Fields] OR “organization and administration”[MeSH Terms] OR (“organization”[All Fields] AND “administration”[All Fields]) OR “organization and administration”[All Fields] OR “management”[All Fields] OR “disease management”[MeSH Terms] OR (“disease”[All Fields] AND “management”[All Fields]) OR “disease management”[All Fields]))	2142	11	3
**Web of Sciences**	Results for ((ALL=(care stress)) AND ALL=(stress management)) AND ALL=(nurse) and Article (Document Types) and English (Languages) and Article (Document Types)	1671	21	15
**Scopus**	TITLE-ABS-KEY (care stress OR care OR stress) AND TITLE-ABS-KEY (“nurse” AND stress management) AND (LIMIT-TO (DOCTYPE,”ar”))	1840	5	3
**SID**	(care stress) AND (stress management) AND (“nurse”)	953	3	1

*Based on title and abstract; **According to inclusion and exclusion criteria.

**Figure 1:**
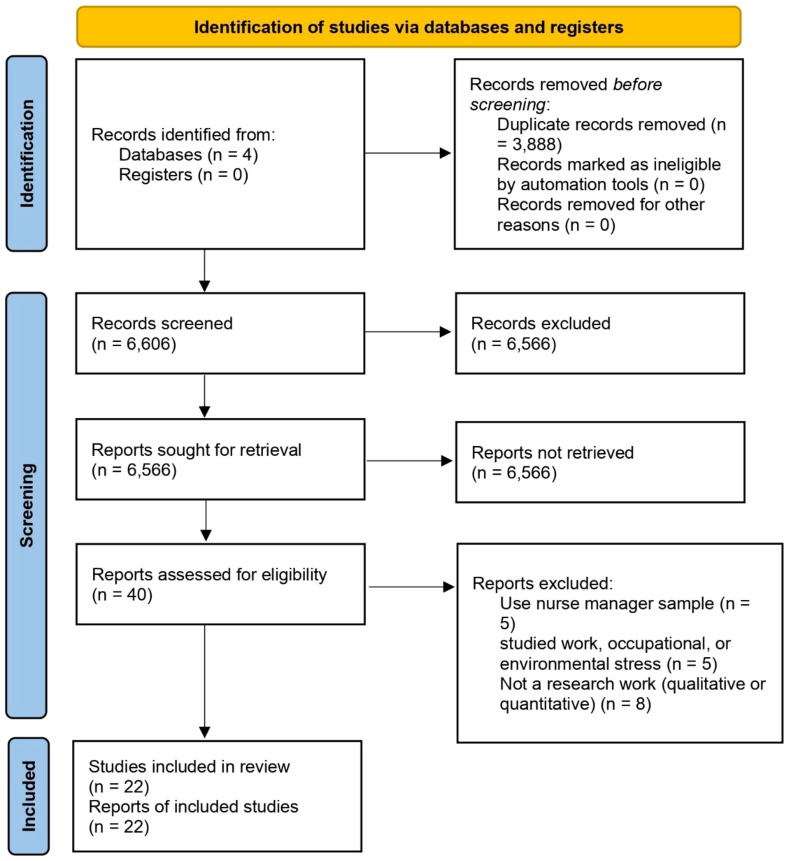
Prisma diagram.

First, duplicate studies from the initial search were removed after selected studies were entered into the Endnote software. Two researchers with doctoral degrees (AHG and EN) obtained original articles and then carefully evaluated them for inclusion. Studies needed to meet the following criteria for inclusion: 1) use nursing population; 2) use caring stress concept or stress management in title or abstract; and 3) published in English and Farsi languages. Studies that 1) studied job, occupational, or environmental stress; 2) had an insufficient sample size (<50 participants) exception for pilot and qualitative studies [because of small power, but pilot and qualitative studies typically commence with smaller sample sizes (30)] 3) were unable to download the full text of the article; 4) were written in languages other than English or Farsi were excluded; and 5) systematic reviews/meta-analyses. Grey literatures (e.g., dissertations, conference proceedings, reports) were not searched. The phases of article selection were based on PRISMA guidelines (version 2020) and are shown in **Figure 1** (31).

### 2.4 Data extraction

Two reviewers conducted independent data collection from full-text papers, ensuring that each paper was assessed by at least two reviewers. The collected data was then compiled for reporting using a structured data extraction process. This extracted information included publication details, author information, research methods, study population, the main concept being investigated, contextual information, and the key findings.

## 3 Results

Out of a total of 104,094 articles initially searched, 22 articles were included in this study based on the defined criteria (6, 12–14, 32–49). **Table 1** and **Figure 1** provide detailed information on the stages of article search.

### 3.1 Characterizes of articles

Based on what is observable in **Table 2**, the studies were conducted in two main categories: cross-sectional and qualitative. The geographical distribution of the studies was diverse and comprehensive, with articles selected from Australia (n = 1), Brunei (n = 1), Brazil (n = 2), Canada (n = 1), China (n = 2), England (n = 1), France (n = 1), Iran (n = 4), Jordan (n = 1), Malaysia (n = 1), Poland (n = 1), South Korea (n = 1), South Africa (n = 1), Taiwan (n = 2), United States (n = 2).

**Table 2:** Main information of articles about care stress of nurses.

Author (year)	Study type	Place of study	Sample size	Scale	Mean age or Age range	Main findings
**Happell, Dwyer** (13)	Qualitative exploratory	Australia	38	Interview	Not reported	High workloads, unavailability of doctors, unsupportive management, human resource issues (incorrect payments), interpersonal issues (bullying, communication problems and conflict between nurses), Relatives of patients, shift work, car parking, handover procedures, no common area for nurses, mental health issues were common cause of stress in nurses. Also, workload modification, leadership within the ward, changing shift hours, ensuring nurses get breaks, music in wards, organizational development, massage therapists on the wards are the main ways of stress management.
**Cavalheiro, Moura Junior** (9)	Cross-sectional	Brazil	75	Researcher made	23 to 47 years	There was no relationship between working shifts and marital status with stress. But direct relation was founded between individual differences, situations at work, and changes in sleep and wake with stress.
**Hamaideh and Ammouri** (33)	Cross-sectional	Jordan	464	Nursing stress scale	25 to 44 years	Inadequate preparation, lack of support, uncertainty concerning treatment, conflict with other nurses, conflict with other physicians were identified as stressors for nurses. Also, death and dying and workload are the common cause of stress among all groups of nurses.
**Hosseini Moghaddam, Mohebbi** (14)	Qualitative content analysis	Iran	16	Interview	25 to 48 years	Concern over transmitting the infection to one’s family, fear of the unknown aspects of the disease, concern over making wrong decisions, families’ insistence on quitting one’s job, working in difficult conditions, lack of personal protective equipment, feeling rejected by the personnel were the main causes of stress. Effective communication skills, experiencing personal-professional growth (improved learning), perception of positive feelings at the end of a crisis were the main ways of stress management.
**Li, Liu** (12)	Exploratory study	Taiwan	792	Nurse Stress Checklist	17 to 46 years	The most frequent areas associated with stress were: the participant cannot finish what s/he wants to do in terms of nursing work, the participant has limited time to complete his/her tasks properly, the participant needs time to interact with family and friends. In addition, caring behavior was significantly and negatively associated with stress.
**Vahedian-Azimi, Hajiesmaeili** (34)	Cross-sectional	Iran	100	Stressful Situations Instrument	29.4 years	levels of collaboration, working with a supervisor on the unit, and nurse–patient ratios were all positively associated with greater stress levels.
**Harvey and Tapp** (35)	Qualitative	Canada	9	Interview	34–58 years	Moral distress (stopping resuscitation), the unspoken ‘Unwritten rules’ of the ICU (conceal emotions about patients for not being judged), tampering with the human connection (the restrained emotions that may be experienced in autopilot may compromise the human connection and expert practice that are necessary in the care of the critically ill) were main reasons of stress. Also, relating and connecting, transformation as an altered professional identity (the change in ‘how I nursed) can support them.
**Ibrahim, Isa** (36)	Cross-sectional	Brunei	113	Spiritual Coping Questionnaire	30–39 years	Religious activities such as finding relief in prayer, focus on higher power, and feel the presence of God in everyday life was the main supportive action for preventing stress. Achieve inner peace, positive thinking, and selfcare were personal factors of stress management.
**Pan, Wang** (37)	Qualitative	China	33	Interview	28.33 years	Concern about occupational exposure (risk of contracting HIV infection), heavy workload, patients’ mental health problems and aggressive behaviors, and perceived discrimination from families and colleagues. Positive coping (using personality strengths, using problem-solving skills, seeking help), and negative coping (concealing work place, avoiding/suppression emotions) were the main ways of stress management.
**Vioulac, Aubree** (38)	Qualitative	France	23	Interview	40.9 years	Time management in providing care, role of emergencies and nurses’ experience, high technicality of specific HD treatment were related with stress of nurses.
**Callaghan, Tak‐Ying** (39)	Cross-sectional	China	500	Anxiety Stress Questionnaire	22 to 44 years	Nursing issues (work overload, dealing with emergencies, responsibilities inherent in job), interpersonal relationships (dealing with patients and relatives, relationships with colleagues, dealing with ward managers and supervisors), hospital administration (inadequate staff and overcrowded ward), duty issues (working night duty and working overtime), ward/clinic management issues (unsupportive seniors and frequent changes of departmental policy), promotion and career development issues (poor prospect for promotion, and taking part in staff development reviews), doctor-related (dealing with doctors, ward rounds, poor attitude of doctors) were the main factors of stress.
**Okunogbe, Meredith** (40)	Cross-sectional	USA	272	Checklist	26 to 45 years	Proportion of high-risk patients, provider satisfaction with help received, care coordination time were the main factors related to stress.
**Siqueira, Teixeira** (41)	Qualitative	Brazil	20	Interview	33 to 54 years	Empathy and projection of the other’s disease itself, causing sadness, and the absence of the other, due to illness, which generates work overload, that is, we have physical and psychic influences. The working conditions include physical, chemical and biological issues of the work organization, as well as aspects related to the health and safety of the professional.
**Kowalczuk, Shpakou** (42)	Cross-sectional	Poland	284	Mini-Cope inventory	Not reported	Active strategies (active coping, planning, positive reframing, acceptance, humor, religion, use of emotional support, use of instrumental support, self-distraction, denial, and venting), and avoidance strategies (substance use, behavioral disengagement, and self-blame) were stress management strategies.
**Huang and Yu** (6)	Cross-sectional	Taiwan	–	Researcher made	Not reported	Patient care (limitations for patients in ICU, workload, unfamiliarity with infectious diseases, inconsistency in operating standards, and not familiar with the samples), infection protection, and support system are the main reasons of stress. *
**Shim and Jeong** (43)	Cross-sectional	South Korea	178	Psychosocial Well-Being Index Short Form	41.68 years	Educational history and past experiences were the main factors that related with stress.
**Beng, Chin** (44)	Qualitative	Malaysia	20	Interview	30-40 years	Organizational challenges, care overload, communication challenges, differences in opinion, misperceptions and misconceptions, personal expectations, emotional involvement, death and dying thoughts, and appraisal and coping were the sources of stress. *
**Rouhbakhsh, Badrfam** (45)	Qualitative	Iran	20	Interview	35.5 years	The inconsistency in medical information, the obscurity of the disease, the likelihood of transmission to the family, unforeseen exposure to infectious droplets, the likelihood of getting infected, being shocked in the early days, resource allocation were the main reasons of stress. Also, visiting patients who have recovered, paying attention to the positive aspects of covid-19, hoping not to get infected, self- reassurance due to personal care, reducing the stress by observing the downward trend of the disease were protective factors against stress.
**Garcia and Marziale** (46)	Cross-sectional	Brazil	122	Researcher made	25 to 66 years	Lack of organizational information, insufficient time due to work overload, lack of clarity in the distribution of tasks and ineffectiveness in interpersonal communication were the main reasons of stress.
**Lipp and Fothergill** (47)	Qualitative	UK	12	Interview	Not reported	Decision to undergo an abortion (nurse must facilitate but not unduly influence the decision in the face of her own views), procedure, medical abortion, suspending judgement (being non-judgmental is a probable stressor), moral distress were the main reasons of stress. Also, personal approaches included personal qualities, being able to relax, having confidence and belief in one’s own abilities, having a sense of humor, and social support were effective ways to cope with stress.
**Schoombee, van der Merwe** (48)	Qualitative	South Africa	8	Interview	Not reported	Nurses’ roles as caregivers within the context of the hospital, work environment (high workload, and lack of resources), resistant patients (don’t obey), hospital hierarchies (staffs and patients), aggressive feelings, thoughts and actions between nurse and patient.
**Hajiseyedrezaei, Alaee** (49)	Cross-sectional	Iran	235	Expanded Nursing Stress Scale	Not reported	Difficulties in relationship between co-workers and nurse managers, death and dying, uncertainties about effectiveness of treatments, patients and their families, emotional unpreparedness were the most common reasons of stress among nurses.

The qualitative studies had sample sizes ranging from 8 (48) to 38 nurses (13), while the quantitative studies ranged from 75 (9) to 792 nurses (12). The age range of the nurses included in these studies was between 17 (12) and 66 years (46). Furthermore, all the studies used various tools to assess stress in nurses. Among these tools, the Nursing Stress Scale (33), Nurse Stress Checklist (12), and the Expanded Nursing Stress Scale (49) were more specialized for evaluating nurses’ stress.

### 3.2 High workloads

Based on the summary of studies, one of the most notable and commonplace stressors among nurses is workload. Multiple researchers (as indicated in **Table 1**) have pointed out factors such as “Time management in providing care” (37–39, 46), “care coordination time” (13, 33, 40, 44), and “the participant cannot finish what is assigned to do in terms of nursing work” (6, 12, 41). These factors refer to the core concept of “High workloads.” High workload results from various reasons, including a shortage of nurses, a large number of patients, and a lack of medical equipment (such as face mask, and latex gloves) that could facilitate nursing tasks.

In a study, a researcher conveyed from participants, *“It’s the staffing shortages and the skill mix shortages … people still keep coming in the doors, irrespective of what [the] workload is … There’s very little downtime for nursing staff anymore.…It’s fast paced activity. So that’s a major stressor.”* (13). Or in another study a nurse revealed *“…When the epidemic started, we didn’t have access to special gear for COVID-19 protection and had to care for the infected with minimum equipment, in regular masks and uniforms …”* (14).

However, it’s important to highlight that a significant portion of the nurse’s workload is due to the multiple needs of patients and a large number of patients, which can be considered as caring stress (13, 37). When faced with excessive work pressure, nurses may struggle to perform their duties correctly, follow principles, and respond promptly to the patient’s needs (13). High workloads can lead to decreased motivation, reduced quality of work life, increased risk of nursing errors, and patient harm (44).

### 3.3 Transmitting the infection

One of the crucial and often overlooked aspects of nursing is the risk of disease transmission. Multiple researchers have pointed out factors such as the “risk of contracting HIV infection” (37), “infection protection” (6), “likelihood of transmission to the family” (14, 45), “unforeseen exposure to infectious droplets” (45), and “likelihood of getting infected” (45). The risk of contracting a disease is one of the most persistent stressors related to patient care. The risk of infection threatens the nurse and their family. In a study, a researcher conveyed from a nurse, *“…I was really worried that I could be the source of infection to my 60-year-old parents …”*

Despite of the precautionary measures (50), this factor is particularly stressful for nurses working in special care units and for patients with diseases such as HIV and HBV (37). The stress resulting from the fear of infection can lead to nurses avoiding close contact with patients, increasing the likelihood of errors in patient assessment and reducing the quality of care provided (14).

### 3.4 Stress-producing thoughts

Nurses experience situations at various intervals that deeply affect their emotions. “Death and dying” is one of the circumstances that nurses frequently experience as a form of caring stress in hospitals. Witnessing a patient’s death can lead to grief, sorrow, and even a sense of the illness affecting the nurse personally (33). It may also instill the thought that a similar fate could befall other patients, which can be a continuous source of stress for nurses (44). In a study, a researcher conveyed from a nurse, *“Last 2 days, patient was alright. He could talk. And then suddenly he is dying. Yesterday, I talked to him he was very happy … Because I have many medical problems as well, I think maybe I can also be like that. I have to be aware, like today we are happy, we are still alive, I can do my work, and then tomorrow, I can change very fast”* (44).

Another aspect of stressful thoughts is the lack of sufficient knowledge and awareness about a disease (14). A knowledge gap regarding a disease, its stages, and transmission methods can lead to stress among nurses and, ultimately, the potential for inadequate care delivery. Additionally, the fear of providing appropriate and quality care and making incorrect decisions related to patient care is another factor related to caring stress (49). Similar to other healthcare professionals, nurses, due to their high workload, are at risk of making errors in decision-making and prioritizing decisions related to the nursing process (14). However, under certain conditions, this risk can constantly occupy a nurse’s mind and lead to caring stress.

However, one of the challenging dimensions is the moral stressors in patient care (35, 47). Nurses consistently encounter a range of challenges that may exert ethical pressure on them. Ethical challenges such as Euthanasia, patient requests for do-not-resuscitate (DNR) orders, conflicts between the patient’s care needs, the nurse’s desires, and the organization’s requirements are among the most significant ethical challenges that nurses encounter (47). Harvey and Tapp (35) conveyed from a nurse, *“I almost begged the resident physician to stop the resuscitation; everyone wanted him to stop. Why is he not stopping this? Why are you making us do this? I don’t want to do this anymore because the patient is so clearly dead. In my career as an ICU nurse, I have learned that I want to respect you in life and in death”.*

### 3.5 Stress-producing emotions

One of the significant issues that hospital staff, especially nurses, often face is patient violence towards healthcare providers (37, 48). This problem is particularly prevalent in emergency departments. These individuals are vulnerable to psychological and physical harm, making them susceptible to stress, especially in such situations. Some nurses may not be emotionally prepared to deal with these conditions in a hospital, and this lack of readiness can also increase the potential for stress among nurses (48).

Another aspect to consider is the nurse’s satisfaction in patient care (40). Nurses feel satisfied after providing care and witnessing the improvement in the patient’s condition. However, it’s possible that this situation could develop into a recurring pattern of obsessive thoughts for the nurse, ultimately resulting in caring stress (44). In a study, a researcher conveyed from a nurse, *“Sometimes I feel sad because I can’t help all of them. Even though we have only 8 patients, I can’t see them for the whole day … I feel stressed when I can’t help them to fulfill their needs…. What I do is I just avoid her. I can’t help her. I’ve tried my best but I can help her much. It’s difficult”* (44).

Also, being judged by the patient can create potential stress in the care process (47). A suitable therapeutic relationship, devoid of judgment, from the nurse to the patient and vice versa, can cultivate a positive, stress-free connection, thereby contributing to the patient’s well-being.

### 3.6 Stressful communications

Nurses engage in more direct interactions with patients in a hospital setting compared to other healthcare professionals (46), and these interactions can occasionally be stressful for the nurse due to a multitude of reasons (44). In emergency departments, where patient families are often present, sometimes in significant numbers, alongside the patient, it can be stressful for nurses and even lead to care disruptions (39). This is according to a nurse’s statement in a study, *“Some relatives can be very fussy. ‘‘Oh, nurse, how is the situation? How about this one?’’ or ‘‘Nurse, why is it like that?’’ They call us and ask, ‘‘Why? Why? Why?’’ on everything! We explain to them but they are still not satisfied. They still can’t understand”* (44).

It’s worth noting that patients who do not follow the nurse’s instructions and recommendations can also cause stress for the nurse. Non-compliance with nursing instructions by the patient can be perceived as a threat to the patient’s health, and the nurse, as the person responsible and accountable for the patient’s well-being, may view it as a threat (48). Therefore, this can lead to nurses experiencing caring stress. Schoombee, van der Merwe (48) said *“sometimes, because when the patient doesn’t cooperate, you as a sister or a nurse know what the consequences could be when they don’t cooperate”.*

Furthermore, interactions with patients in need of emergency care, such as those with extensive bleeding or cardiac resuscitation, can be highly stressful (39). Nurses unaware of the patient’s condition and status are more susceptible to stress than others (6). For example, a newly hired nurse or a nurse receiving a new patient may experience more stress than other situations.

It’s worth noting that the handover procedure has been identified as a stressor in the review of a study (13). This process, which occurs at the end of a work shift to transfer the patient to another nurse, is crucial because it involves the safe and effective transfer of patient care and their condition. However, it can sometimes lead to stress, especially in less experienced nurses, as it is a sensitive and vital aspect of patient care (51).

### 3.7 Other stressful events

Among other extracted factors, researchers have highlighted the role of a nurse’s experience level (38), the development of advanced equipment and devices in patient care (sometimes the use of these equipment can be challenging and stressful for nurses) (38), the nurse’s responsibilities and accountability (39), limitations impacting patient care in critical care units (such as complexity of cases, and emotional impact) (6), and inconsistencies in patient information and medical histories (45). These factors can all contribute to the stress experienced by nurses in their roles.

### 3.8 Caring stress management

Researchers have proposed multiple strategies for managing caring stress, as outlined in **Table 2**. These strategies include considering rest breaks during patient care shifts (13), playing music in the ward (13), providing education and enhancing therapeutic communication skills for nurses, personal and professional growth efforts, promoting a positive mindset at the end of a crisis or adverse event (14), improving problem-solving skills, seeking assistance from colleagues during stressful situations (37), embracing critical or stressful situations and working towards appropriate change (42), humor (47), using religion or spirituality to reduce stress, proper and effective use of medical devises to aid nursing activities (42), self-distraction (42), increasing encounters with patients who have recovered (which alleviates stress by observing a declining trend in the disease) (45), fostering optimism that the disease won’t transmit to the nurse (45), having self-confidence and belief in personal abilities, and enhancing social support (47). Additionally, strategies such as emotion suppression (37), denial of critical situations, substance use, and self-blame (42) were mentioned as negative coping mechanisms.

## 4 Discussion

According to the results of the present study, several factors can elevate the risk of caring stress among nurses.

### 4.1 Care workloads

Many previous studies have indicated that workload has a significant effect on caring stress (6, 13, 33, 37, 48). High workloads significantly impact nurses’ stress levels. The constant pressure to meet patient care demands, manage administrative tasks, and handle the emotional toll of nursing can lead to intense stress and strain.

This high stress can take a toll on both their physical and emotional well-being. Nurses often struggle to balance work and personal life, which further adds to their stress (37). Furthermore, nurses may find it challenging to maintain the same level of care, which can result in reduced quality of patient care and cause additional stress as they worry about patient outcomes (52).

High workloads are a primary factor contributing to nurse burnout, characterized by extreme stress, cynicism, and decreased job satisfaction. Burnout exacerbates the stress experienced by nurses, making it a critical concern for healthcare organizations (53). High workloads frequently lead to moral distress among nurses, as they are unable to deliver the planned care to their patients. This moral distress intensifies their stress and may erode their sense of purpose in the nursing profession (35).

The stress resulting from high workloads can have adverse health consequences for nurses, including sleep disturbances, an increased susceptibility to chronic illnesses, and mental health challenges. Therefore, addressing the stress of nurses due to high workloads is crucial for the well-being of healthcare professionals and directly affects the quality of patient care (54). Healthcare organizations must take proactive measures to mitigate these stressors, foster a supportive work environment, and prioritize the mental and physical well-being of their nursing staff.

### 4.2 Transmitting infections

Transmitting infections in a healthcare setting places immense stress on nurses. Previous research findings have confirmed that the fear of transmitting infections plays a significant role in contributing to caring stress among nurses (6, 14, 37). There were no contradictory results about the effect of fear of transmitting the infection. The fear of transmitting infections significantly impacts the caring stress experienced by nurses, manifesting in various ways within their professional and personal lives. This fear often stems from the inherent risk of exposure to infectious diseases in healthcare settings, especially during pandemics or outbreaks.

Constant vigilance to adhere to infection control protocols, including wearing personal protective equipment (PPE) and maintaining strict hygiene measures, adds layer of stress to their already demanding roles (55). Furthermore, the fear of unknowingly transmitting infections to vulnerable patients or loved ones outside of work can cause emotional strain and heightened anxiety among nurses. This fear affects their mental well-being and influences their decision-making processes and interactions with patients, potentially leading to burnout and decreased quality of care (6).

The constant fear of contracting or spreading infectious diseases creates a challenging work environment. Strict adherence to infection control measures, including wearing personal protective equipment and practicing rigorous hand hygiene, adds to the mental and physical burden (14). The emotional toll of witnessing patients’ suffering and the potential loss of life intensifies the stress. Isolation from patients and their families, combined with ethical dilemmas and heightened workloads, exacerbates the stress endured by nurses. Additionally, the personal risk nurses may pose to their families when returning home after caring for infectious patients adds to their anxiety (45).

To alleviate this stress, healthcare organizations should provide comprehensive support, including education and training, mental health resources, effective communication, and providing resources and personal protective equipment. It’s crucial to recognize and address the stressors nurses face in dealing with the transmission of infections and to prioritize their well-being in these challenging situations (56).

### 4.3 Stressful thoughts

Researchers have unveiled that the fear of patient death, encounters with patients having complex or rare medical conditions, and decisions related to patient care are significant factors contributing to the caring stress experienced by nurses (14, 33, 44, 47, 49).

The fear of patient death is a profound emotional source of caring stress for healthcare professionals, particularly nurses. Healthcare providers often develop strong emotional bonds with their patients, making the fear of losing a patient a highly distressing experience (49). The emotional attachment they form can foster empathy and compassion but also exposes them to the emotional toll of witnessing patient suffering and, in some cases, death. Patient deaths, especially when they are unexpected or perceived as premature, can trigger feelings of grief and loss among nurses.

The sadness, despair, and profound sense of emptiness accompanying such losses can accumulate over time. In settings where healthcare professionals encounter frequent deaths, this accumulation of grief can lead to a form of cumulative stress, known as “compassion fatigue” (57). Furthermore, when healthcare professionals are unable to prevent patient deaths or provide the level of care, they believe their patients deserve due to factors beyond their control, they may experience moral distress. Moral distress arises when one’s values and ethical principles are compromised, creating internal conflict and a sense of powerlessness. This internal struggle amplifies the emotional stress experienced by healthcare professionals.

Nurses often encounter patients with complex and rare medical conditions. When they lack sufficient knowledge and awareness about a specific disease, it can lead to feelings of insecurity and anxiety. They may experience fear of making mistakes or providing suboptimal care (14). The need to quickly acquire knowledge about an unfamiliar disease or condition can be overwhelming, especially when nurses are already dealing with a heavy workload. This added pressure can intensify their stress (58). On the other hand, ethical stressors frequently emerge when nurses are faced with challenging decisions concerning patient care. They may face situations where they should balance patient autonomy, beneficence, and justice, which can create moral dilemmas (59). Ethical conflicts can lead to moral distress when nurses feel unable to act in a way that aligns with their moral and ethical values. This distress can be emotionally draining and contribute to caring stress (35).

Healthcare organizations should recognize and validate the emotional challenges nurses face. Offering access to counseling services, debriefing sessions, and peer support can help nurses cope with their emotional stress (42). Ensuring manageable workloads and staffing levels can reduce the emotional burden on nurses. Adequate staffing allows nurses to focus on providing quality care rather than feeling overwhelmed by excessive responsibilities.

### 4.4 Stressful emotions

Another significant factor influencing caring stress, unanimously agreed upon by all researchers, is aggressive behavior from patients and the necessity to provide care without judgment (37, 47, 48). Aggressive behavior exhibited by patients, such as verbal outbursts, threats, physical aggression, or agitation, can create a challenging and often hostile work environment for nurses. These situations can be unpredictable and require immediate and effective responses. The safety of both the nurse and the patient is a significant concern when dealing with aggressive behavior (37).

Nurses may harbor concerns for their safety, as well as the safety of their colleagues and other patients. This constant fear can lead to high levels of stress. Coping with the aggression of patients can take a severe emotional toll on nurses. They may experience various emotions, including fear, frustration, anger, helplessness, and even sadness (60). These emotions can accumulate over time, contributing to caring stress. Nurses often require training in de-escalation techniques and conflict resolution to effectively manage aggressive patient behavior. Having the skills to defuse potentially volatile situations can help reduce the emotional impact of dealing with such behavior.

One of the core principles of nursing is to provide care without judgment. Nurses are ethically and professionally bound to treat all patients respectfully and without bias, regardless of their backgrounds, beliefs, or lifestyles (47). In healthcare, nurses encounter patients with diverse and complex medical histories. These histories may include substance use disorders, past criminal activity, or unconventional lifestyles. The need to withhold judgment while caring for such patients can be challenging. Balancing the commitment to non-judgmental care with personal values and beliefs can take an emotional toll on nurses. They may grapple with their own biases, prejudices, or discomfort when caring for patients whose lifestyles or choices they do not fully understand or agree with (61).

Training in de-escalation techniques and strategies for handling aggressive patient behavior can help nurses to reduce stress. Equip nurses with the skills to defuse tense situations and cope with the emotional impact of aggression. Encourage a non-judgmental and inclusive organizational culture that respects diversity and emphasizes providing care without bias (47). Ensure that nurses have access to support systems and resources, such as counseling services, peer support, and debriefing sessions, to help them manage the emotional challenges of their work (37).

### 4.5 Stressful communications

Distressing news to patients and their relatives, providing medical advice to patients, and encountering life-threatening situations are among the main stress-inducing communications that many researchers assert have a significant impact on the caring stress experienced by nurses (9, 13, 34, 36, 39).

Nursing often involves delivering difficult or distressing news to patients and their relatives. Discussions about terminal illnesses, end-of-life care decisions, or adverse treatment outcomes can be emotionally draining for nurses. They may absorb the emotional burden of these conversations, leading to feelings of sadness and empathy fatigue (39). Stressful interactions with patients or their relatives may arise from disagreements, misunderstandings, unrealistic expectations, or emotional distress. Nurses should often act as mediators, addressing conflicts and maintaining a calm and professional demeanor. This role can be challenging and contribute to their overall stress (62). Nurses are expected to provide care with empathy and compassion, yet they must also maintain professionalism. Balancing these aspects can be demanding, requiring them to show understanding and support while adhering to clinical guidelines and maintaining emotional detachment (63). This emotional labor can add to their stress.

Nurses often invest considerable time and effort in providing patients with medical advice and treatment plans. When patients do not comply with these recommendations, nurses may experience frustration and concern. They may fear non-compliance could lead to worsened health outcomes or complications (48). Educating patients about their conditions, treatment options, and the importance of following medical advice is a fundamental aspect of nursing care. However, nurses may encounter challenges in patient education, such as language barriers, low health literacy, or resistance to accepting the advice given (64). Nurses often develop a strong sense of responsibility for their patients’ well-being. When patients do not adhere to medical advice, nurses may worry about the potential negative consequences. This concern for patient outcomes can be a significant source of stress.

Life-threatening situations, such as cardiac arrests, respiratory distress, or trauma cases, require nurses to make rapid, critical decisions. The urgency and high-stakes nature of these situations can lead to acute stress. Nurses must act quickly and efficiently to provide life-saving care, leaving little time for reflection (39). Life-threatening situations often involve intense emotions, such as fear, anxiety, and the need to comfort distressed patients and their families. Nurses may absorb these emotions while striving to maintain a calm and reassuring presence. Coping with the emotional intensity of these situations can be emotionally exhausting (39). Nurses who are regularly exposed to traumatic events, such as unsuccessful resuscitation attempts, multiple patient deaths, or severe injuries, are at risk of developing symptoms of post-traumatic stress disorder (PTSD). The emotional toll of witnessing life-threatening situations can lead to long-term psychological distress, further intensifying caring stress (65).

Communication and conflict resolution training can help nurses handle difficult conversations with patients and their families more effectively, reducing the emotional burden (42). Nurses should have access to debriefing sessions and emotional support services to help them process the stress and emotions related to challenging patient interactions and life-threatening situations (14). Healthcare organizations can develop patient education resources that are easily understandable, culturally sensitive, and designed to promote compliance with medical advice.

### 4.6 Cultural factors

Based on the findings, studies from a diverse range of countries were included. Cultural factors play a pivotal role in shaping the perceptions of caring stress and the coping mechanisms nurses utilize. One key aspect is the cultural definition and interpretation of caring stress itself. Cultural norms and expectations regarding work, family, and societal roles can profoundly influence how nurses perceive and label stressors in their professional and personal lives (66). For instance, in some cultures, there may be a strong emphasis on individual resilience and stoicism, leading nurses to downplay or internalize their stressors rather than seek external support.

Additionally, cultural beliefs about health, illness, and the role of healthcare professionals may impact how nurses perceive their caring stressors. Furthermore, cultural differences in communication styles and social support networks can influence the coping strategies adopted by nurses (46). Some cultures may prioritize seeking support from family and community networks, while others may prioritize seeking support from colleagues or formal mental health services. Moreover, cultural values related to hierarchy and authority within healthcare settings may affect nurses’ willingness to seek help or express vulnerability (36).

Another significant dimension in cultural factors is the presence of migrated nurses. The experiences of migrated nurses within the framework of cultural factors significantly influence the manifestation and management of caring stress in clinical settings (67). Cultural differences in caregiving norms, communication styles, and patient-provider interactions can exacerbate the challenges faced by migrated nurses, leading to heightened levels of caring stress. Migrated nurses may encounter unfamiliar cultural practices, language barriers, and differing patient expectations, which can contribute to feelings of uncertainty and frustration in their caregiving roles (68).

Additionally, acculturative stressors such as adapting to new work environments, navigating complex healthcare systems, and experiencing discrimination further compound caring stress among migrated nurses (67). Understanding and addressing these cultural dynamics are essential for implementing effective strategies to support migrated nurses in managing caring stress and promoting their overall well-being in diverse healthcare settings (69). Overall, by acknowledging and comprehending these cultural influences, healthcare organizations can enhance their support for nurses in managing stress and fostering their well-being.

### 4.7 Caring stress and COVID-19

The pandemic has exponentially increased the workload and complexity of care, leading to heightened physical, emotional, and psychological strain among nursing professionals. With limited resources, ever-changing protocols, and the constant fear of exposure to the virus, nurses have grappled with unprecedented challenges in delivering quality care while safeguarding their well-being (70). The continual exposure to suffering, loss, and trauma has contributed to a surge in compassion fatigue and burnout, further exacerbating the toll on their mental health. Despite these adversities, nurses have demonstrated unparalleled resilience, dedication, and compassion in the face of adversity, underscoring their invaluable role as frontline heroes in the battle against COVID-19 (71).

### 4.8 Limitations and strength

The scoping review may be limited to studies published in a specific language or from particular geographic regions. This could exclude valuable research from other linguistic or cultural contexts, leading to language bias. Scoping reviews may not systematically search gray literature sources, such as conference abstracts, theses, or government reports, which could contain relevant information. This could lead to a publication bias. Findings from this study may not be generalizable to all nursing contexts, as they are influenced by the specific studies included in the review. The diversity of healthcare settings, cultural factors, and nurse populations can limit the external validity of the findings.

It’s worth noting that there has been limited qualitative and quantitative research in nursing caring stress and its management. This is the first study that has focused solely on a review of care stressors. As more studies are conducted worldwide on this topic, it becomes possible to expand the evidence base and perform more in-depth reviews. This will help further our understanding of the specific stressors that nurses face and the most effective strategies for managing and mitigating those stressors in the healthcare setting.

### 4.9 Implications in clinical practice and policy changes

Effective stress management strategies can lead to better patient care and safety. Stressed nurses are more likely to make errors or become less vigilant in their duties, impacting patient outcomes. By addressing caring stress, clinical practice can prioritize patient well-being. Burnout is a significant concern among healthcare professionals, including nurses. High levels of caring stress contribute to burnout and can lead to staff turnover. Stress management techniques can help retain experienced nurses and reduce the costs associated with recruiting and training new staff.

Supporting nurses in managing caring stress is essential for patient care and effective for well-being of healthcare professionals. This can increase job satisfaction, mental health, and overall quality of life for nurses. Stress can impact team dynamics and communication within healthcare teams. By addressing caring stress and providing support, clinical practice can foster a more collaborative and supportive work environment, which is crucial for effective patient care.

Policy changes should advocate for the implementation of recognition and reward systems that acknowledge the efforts of clinical nurses in managing caring stress. Healthcare policies should encourage integrating technology solutions to support nurses in managing caring stress. This may include developing mobile applications for stress tracking and management, virtual support platforms, and telehealth services for accessing mental health support remotely.

According to the results, high workloads, fear of transmitting infections, stressful thoughts, emotional strain, and challenging communications emerged as the major concepts and factors contributing to stress among nurses. Also, some essential ways to manage and diminish stress were presented. Further research is needed to delve deeper into this important issue concerning nurses in the future.

## Data availability statement

The original contributions presented in the study are included in the article/supplementary material. Further inquiries can be directed to the corresponding author.

## Author contributions

AG: Conceptualization, Data curation, Writing – original draft, Writing – review & editing. AN: Methodology, Writing – original draft, Writing – review & editing. HS: Methodology, Writing – original draft, Writing – review & editing. BF: Writing – original draft, Writing – review & editing. EN: Writing – original draft, Writing – review & editing, Methodology, Supervision.
